# Cesarean delivery or induction of labor in pre-labor twin gestations: a secondary analysis of the twin birth study

**DOI:** 10.1186/s12884-020-03369-x

**Published:** 2020-11-17

**Authors:** C Dougan, L Gotha, N Melamed, A Aviram, EV Asztalos, S Anabusi, AR Willan, JFR Barrett, E Mei-Dan

**Affiliations:** 1 Division of Maternal-Fetal Medicine, Department of Obstetrics and Gynecology and the Department of Newborn & Developmental Paediatrics, Sunnybrook Health Sciences Centre, Toronto, Ontario Canada; 2 Department of Obstetrics and Gynecology, North York General Hospital, Toronto, ON Canada; 3 University of Toronto, Toronto, ON Canada; 4 Child Health Evaluative Sciences, SickKids Research Institute, The Hospital for Sick Children, Toronto, ON Canada

**Keywords:** Twins pregnancy, Cesarean section, Induction of labor

## Abstract

**Background:**

In the Twin Birth Study, women at 32^0/7^–38^6/7^ weeks of gestation, in whom the first twin was in cephalic presentation, were randomized to planned vaginal delivery or cesarean section. The study found no significant differences in neonatal or maternal outcomes in the two planned mode of delivery groups. We aimed to compare neonatal and maternal outcomes of twin gestations without spontaneous onset of labor, who underwent induction of labor or pre-labor cesarean section as the intervention of induction may affect outcomes.

**Methods:**

In this secondary analysis of the Twin Birth Study we compared those who had an induction of labor with those who had a pre-labor cesarean section. The primary outcome was a composite of fetal or neonatal death or serious neonatal morbidity. Secondary outcome was a composite of maternal morbidity and mortality. Trial Registration: NCT00187369.

**Results:**

Of the 2804 women included in the Twin Birth Study, a total of 1347 (48%) women required a delivery before a spontaneous onset of labor occurred: 568 (42%) in the planned vaginal delivery arm and 779 (58%) in the planned cesarean arm. Induction of labor was attempted in 409 (30%), and 938 (70%) had a pre-labor cesarean section. The rate of intrapartum cesarean section in the induction of labor group was 41.3%. The rate of the primary outcome was comparable between the pre-labor cesarean section group and induction of labor group (1.65% vs. 1.97%; *p* = 0.61; OR 0.83; 95% CI 0.43–1.62). The maternal composite outcome was found to be lower with pre-labor cesarean section compared to induction of labor (7.25% vs. 11.25%; *p* = 0.01; OR 0.61; 95% CI 0.41–0.91).

**Conclusion:**

In women with twin gestation between 32^0/7^–38^6/7^ weeks of gestation, induction of labor and pre-labor cesarean section have similar neonatal outcomes. Pre-labor cesarean section is associated with favorable maternal outcomes which differs from the overall Twin Birth Study results. These data may be used to better counsel women with twin gestation who are faced with the decision of interventional delivery.

**Supplementary Information:**

The online version contains supplementary material available at 10.1186/s12884-020-03369-x.

## Background

The prevalence of twin gestations is approximately 3% of all pregnancies. They carry a higher risk profile compared to singleton gestations [1], some of which may necessitate delivery prior to the spontaneous onset of labor, or pre-scheduled elective cesarean section (CS).

In attempt to limit perinatal mortality and other adverse neonatal outcome, elective delivery at 37 to 39 weeks of gestation has been widely recommended in twin gestations [1–6].

There is a paucity of information concerning the outcomes of pre-labor deliveries using induction of labor (IOL) compared to pre-labor CS in twins. Published studies have compared IOL in twin and singleton gestations (rather than IOL in twins versus CS in twins) or limit their analysis to specific subgroups, such as monochorionic twin pregnancies [7–9]. The Twin Birth Study is the largest multicenter, randomized controlled trial, in which women with twin pregnancies between 32^0/7^ and 38^6/7^, with the first twin in cephalic presentation, were randomized to planned vaginal delivery (VD) or planned CS. Results demonstrated that planned VD had similar neonatal and maternal outcomes compared to a planned CS [10]. As the study compared planned modes of delivery, the population included both those who had a spontaneous onset of labor and those who required intervention to achieve delivery by IOL or pre-labor CS (PrlCS). A subsequent secondary analysis of women who had a spontaneous onset of labor also showed no significant difference in neonatal or maternal outcomes between planned VD and CS^11^. When facing the need to counsel a patient with twins who requires pre-labor obstetrical intervention, there is still a knowledge gap as to how this intervention, either through IOL or PrlCS, affects neonatal or maternal outcomes.

This secondary analysis sought to evaluate and compare fetal, neonatal and maternal outcomes in the subset of women who required IOL or PrlCS to better inform women during the counselling process.

## Methods

A total of 106 centers in 25 countries participated in the original Twin Birth Study between December 2003 and April 2011. A full detailed description of the study protocol is available elsewhere [10], but in brief; eligibility for randomization was limited to women with two viable fetuses between 32^0/7^ and 38^6/7^ weeks of gestation, with the first twin in cephalic presentation and estimated fetal weights between 1500 g and 4000 g. Women with two or more previous low-segment CS, vertical uterine incision, mono-amniotic twins or any contraindication to vaginal birth were excluded. IOL or PrlCS were performed for obstetrical or medical indications (e.g. preeclampsia) or electively between 37^5/7^ and 38^6/7^of gestation because evidence suggested that perinatal outcomes would be best during this gestational-age window [10].

Method of IOL, oxytocin augmentation protocol, mode of analgesia and the management of the second twin were at the discretion of the attending obstetrician. Ability to perform a cesarean section within 30 min if necessary was a stipulation of trial participation.

The primary outcome of the Twin Birth Study and of this secondary analysis was a composite of fetal or neonatal mortality or serious neonatal morbidity. Serious neonatal morbidity included the following: Birth trauma; Apgar score of less than 4 at 5 min; seizures before 72 h of age; coma; need for assisted ventilation; confirmed septicemia; necrotizing enterocolitis; pneumoatosis intestinalis; bronchopulmonary dysplasia; grade III or IV intraventricular hemorrhage and cystic periventricular leukomalacia. The secondary outcome was a composite maternal outcome, which was defined as any of the following occurring up to 28 days postpartum: Death; severe hemorrhage (blood loss ≥1500 ml or need for blood transfusion); need for dilation and curettage after delivery; laparotomy; genital tract injury; thromboembolism requiring anticoagulation; systemic infection; major medical life-threatening illness; wound infection, dehiscence or breakdown. Since some morbidities are only related to CS and other to VD, a composite outcome served best to evaluate a “severe morbidity” and to compare between IOL and PrlCS in respect to neonatal and maternal outcome. The full protocol and specific methods of identification of each of these outcomes is detailed in the original manuscript [10].

Utilizing the data from the initial Twin Birth Study, in this secondary analysis we compared neonatal and maternal outcomes in the subgroup of women who required delivery prior to onset of labor. As the aim of the study was to assess the impact of PrlCS and IOL on maternal and neonatal outcomes, we did not follow the original randomization arms and the intention-to-treat analysis approach. Since, faced with the decision of IOL, some women randomized to the planned VD arm chose to undertake CS, we felt an analysis based on actual attempt of labor (IOL) versus no attempt (pre-labor CS) would be more appropriate.

Continuous outcomes were compared between groups using mean ± standard deviations and categorical data are presented as percentages. The unit of analysis was the infant when assessing neonatal outcomes. Generalized estimating equations were used to account for maternal age, parity, previous CS, gestational age at delivery, presentation at delivery, antenatal corticosteroids use and for the correlation between infants from the same pregnancy, and presented as adjusted odds ratio (aOR) and the corresponding 95% confidence interval (95%-CI). Statistical significance was set to 0.05, two-sided. A sub-analysis of women that required a delivery for “gestational-age window” in a twin pregnancy (gestational age between 37^5/7^ weeks and 38^6/7^) was also performed. Lastly, an intention-to-treat analysis according to the original randomization groups of the Twin Birth Study was done for patient who required delivery when not in spontaneous labor. Since this analysis was secondary to a randomized clinical trial, no power calculations were performed. The original study and all secondary analyses were approved by the Research Ethics Board at the Sunnybrook Health Sciences Centre (Trial Registration: NCT00187369).

## Results

In the original Twin Birth Study, 2804 women were included. A total of 1347 (48.0%) women required delivery prior to the onset of spontaneous labor: 779 (57.8%) in the planned CS arm and 568 (42.2%) in the planned VD arm. Overall, 31 women (3.9%) in the planned CS arm and 190 women (33.4%) in the planned VD arm crossed over to the other group following a discussion regarding the need for delivery.

**Fig. 1:**
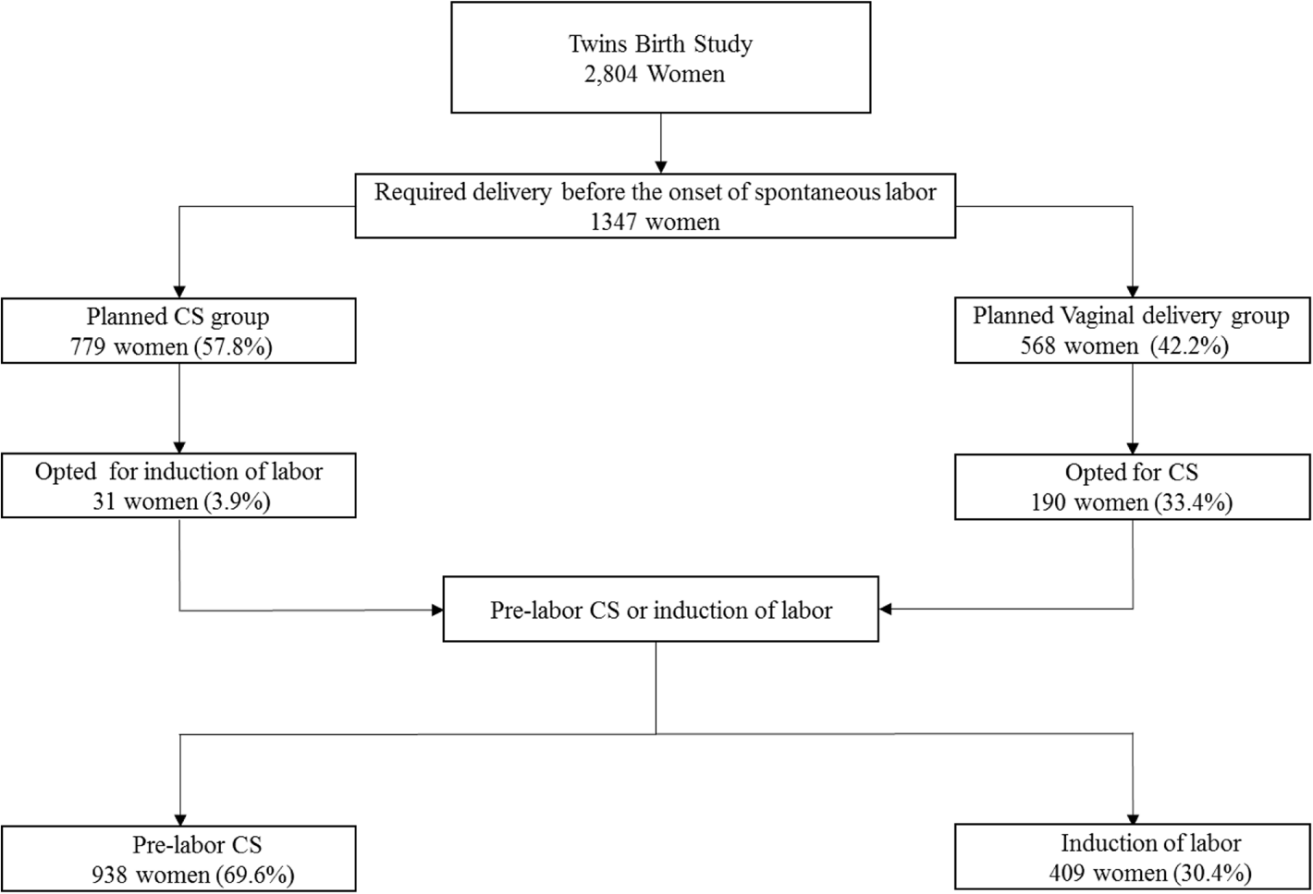
Flow chart of women who underwent pre-labor cesarean section (CS) or induction of labor in the Twin birth study

Of the 1347 women who needed interventional delivery prior to the onset of spontaneous labor, 409 (30.4%) had an IOL and 938 (69.6%) PrlCS (Fig. 1). The groups were significantly different with regards to maternal age, parity, previous cesarean section status, antenatal corticosteroids use, estimated fetal weight of the second twin, and gestational age and presentation at delivery (Table 1). In the IOL group, 155 women (37.9%) had an intrapartum CS for both twins, and 14 (3.4%) had a CS for the second twin.

**Table 1:** Characteristics of women who underwent pre-labor cesarean section (PrlCS) or induction of labor (IOL)

Characteristic n (%)	PrlCS (***n*** = 938)	IOL (***n*** = 409)	***P***
Maternal age (y) mean ± SD	29.7 ± 6.1	30.5 ± 6.1	**0.01**
≥30	445 (47.4)	212 (51.8)	0.14
Parity ≥1	560 (59.7)	208 (50.9)	**0.003**
Previous cesarean section	95 (10.1)	18 (4.4)	**0.0005**
Estimated fetal weight			
First twin (g) mean ± SD	2245 ± 430	2284 ± 420	0.12
Second twin (g) mean ± SD	2225 ± 422	2291 ± 432	**0.008**
**Mode of delivery**			
Vaginal for both	0	240 (58.7)	
Cesarean for both	938 (100.00)	155 (37.9)	**< 0.0001**
Vaginal/cesarean	0	14 (3.4)	
**Presentation at delivery**			
Cephalic/Cephalic	503 (53.6)	276 (67.5)	
Cephalic/Non-cephalic	376 (40.1)	131 (32.0)	**< 0.0001**
First twin in non-cephalic	58 (6.2)	2 (0.5)	
**Gestational age at delivery (wk)**			
Mean ± SD	37.01 ± 1.35	37.51 ± 1.17	**< 0.0001**
32 wk. 0 days to 33 wk. 6 days	30 (3.2)	3 (0.7)	
34 wk. 0 days to 36 wk. 6 days	328 (35.0)	115 (28.1)	
37 wk. 0 days to 38 wk. 6 days	560 (59.7)	270 (66.0)	**< 0.0001**
≥ 39 wk. 0 days	20 (2.1)	21 (5.1)	
Interval between deliveries (min) Mean ± SD	2.2 ± 3.2	12.2 ± 21.2	**< 0.0001**
Use of antenatal corticosteroids	274 (29.2)	78 (19.1)	**< 0.0001**
Chorionicity at birth			
Dichorionic- diamnionic	699 (74.5)	304 (74.3)	0.91
Monochorionic-diamnionic	214 (22.8)	92 (22.5)	
Monochorionic-monoamniotic	1 (0.1)	1 (0.2)	
Undocumented	24 (2.6)	12 (2.9)	

*PrlCS* Pre-labor cesarean section**,** *IOL* Induction of labor

The rate of the primary neonatal outcome was comparable between the PrlCS and IOL groups (1.65% vs. 1.97%; *p* = 0.61; aOR 0.83; 95% CI 0.43–1.62). The rates of the individual components of the neonatal composite outcome were also similar between groups (Table 2).

**Table 2:** Neonatal outcomes in women who underwent pre-labor cesarean section (PrlCS) or induction of labor (IOL)

Outcome n (%)	PrlCS (***n*** = 938)	IOL (***n*** = 409)	aOR (95% CI)	***P***
Composite primary outcome	31 (1.7)	16 (2.0)	0.8 (0.4–1.6)	0.61
Death	15 (0.8)	6 (0.7)	1.1 (0.4–3.3)	0.88
Serious neonatal morbidity^a^	16 (0.9)	10 (1.2)	0.7 (0.3–1.6)	0.4
Birth trauma ^b^	2 (0.1)	2 (0.2)	0.4 (0.0–4.8)	0.49
Apgar score < 4 at 5 min	2 (0.1)	3 (0.4)	0.28 (0.0–1.7)	0.24
Abnormal level of consciousness ^c^	0	0	–	
≥2 Seizures within 72 h after birth	0	2 (0.3)		0.09
Assisted ventilation ^d^	12 (0.7)	2 (0.3)	2.61 (0.6–11.9)	0.13
Neonatal sepsis within 72 h after birth	0	0	–	
Necrotizing enterocolitis	0	1 (0.1)		0.30
Cystic periventricular leukomalacia	2 (0.1)	0		0.99

*PrlCS* Pre-labor cesarean section, *IOL* Induction of labor

Adjusted odds ratio (aOR) and their 95% confidence intervals (95%-CI) represent the result of a generalized estimating equation, accounting for maternal age, parity, previous CS, gestational age at delivery, presentation at delivery, antenatal corticosteroids use and for the correlation between infants from the same pregnancy

^a^Serious neonatal morbidity: ^b^birth trauma (long-bone fracture, other bone fracture, facial-nerve injury at 72 h of age or at discharge, intracerebral hemorrhage); Apgar score < 4 at 5 min; neurological (≥ 2 seizures before 72 h of age; coma; stupor or decreased response to pain); respiratory (assisted ventilation for ≥24 h by endotracheal tube, inserted before 72 h of age; bronchopulmonary dysplasia); neonatal sepsis before 72 h of age; necrotizing enterocolitis; grade III or IV intraventricular hemorrhage and cystic periventricular leukomalacia

^c^Abnormal level of consciousness: Coma, stupor or decreased response to pain, hyperalert, drowsy, or lethargic

^d^Assisted ventilation for ≥24 h by means of endotracheal tube, inserted within 72 h after birth

The maternal composite outcome was significantly lower in the PrlCS group: 7.25% vs. 11.25% (*p* = 0.01; aOR 0.61; 95% CI 0.41–0.91), and this difference stemmed primarily from the differences in hemorrhagic morbidity. Compared with women in the IOL group, women in the PrlCS group had significantly lower rates of hemorrhage (6.19% vs. 9.56%; *p* = 0.02; aOR 0.62; 95% CI 0.40–0.94), and required less postpartum uterine dilatation and curettage (0.32% vs. 1.47%; *p* = 0.03; aOR 0.21; 95% CI 0.05–0.86). The rate of severe perineal injury (3rd or 4th degree) in the IOL group was 0.98%. There were no significant differences between the two groups in respect to other individual maternal outcomes (Table 3).

**Table 3:** Maternal outcomes in women who underwent pre-labor cesarean section (PrlCS) or induction of labor (IOL)

Outcome n (%)	PrlCS (***n*** = 938)	IOL (***n*** = 409)	aOR (95% CI)	***P***
Death or serious maternal morbidity	68 (7.3)	46 (11.3)	0.61 (0.4–0.9)	**0.01**
Death	1 (0.1)	1 (0.2)	0.43 (0.0–7.0)	0.55
Hemorrhage	58 (6.2)	39 (9.6)	0.62 (0.4–0.9)	**0.02**
Blood loss ≥1500 ml	19 (2.0)	14 (3.4)	0.58 (0.3–1.2)	0.13
Blood transfusion	49 (5.2)	29 (7.1)	0.72 (0.4–1.2)	0.17
D&C of uterus after delivery^a^	3 (0.3)	6 (1.5)	0.2 (0.1–0.9)	**0.03**
Laparotomy	9 (1.0)	0 (0)	5.5 (1.10-Inf)	0.07
Genital tract injury^b^	3 (0.3)	0 (0)	1.7 (0.25-Inf)	0.67
Perineal third- or fourth-degree tear involving anal sphincter	0 (0)	4 (0.98)	0.1 (0–0.5)	0.01
Thromboembolism requiring anticoagulant therapy	5 (0.5)	1 (0.3)	2.2 (0.3–18.8)	0.47
Infection, excluding wound infection	16 (1.7)	5 (1.2)	1.40 (0.5–3.9)	0.51
Wound infection^c^	20 (2.1)	4 (1.0)	2.20 (0.7–6.5)	0.15
Wound dehiscence or breakdown	12 (1.3)	3 (0.7)	0.17 (0.5–6.2)	0.38

*PrlCS* Pre-labor cesarean section**,** *IOL* Induction of labor

Adjusted odds ratio (aOR) and their 95% confidence intervals (95%-CI) represent the result of a generalized estimating equation, accounting for maternal age, parity, previous CS, gestational age at delivery, presentation at delivery, antenatal corticosteroids use and for the correlation between infants from the same pregnancy

^a^D&C- Dilation and curettage

^b^Genital tract injury: Need for hysterectomy; vulvar or perineal hematoma requiring evacuation; broad-ligament hematoma confirmed by means of ultrasonography, CT, or MRI; intraoperative damage to the bladder, ureter, or bowel requiring repair; fistula involving the genital tract

^c^Wound infection: Infection requiring prolongation of hospital stay, infection requiring readmission to hospital, infection requiring repeated treatment as an outpatient

An intention-to-treat analysis according to the original randomization groups of the Twin Birth Study for patient who required delivery when not in spontaneous labor found no differences in neonatal or maternal outcomes in the two planned mode of delivery groups (Table 4). We also performed a sub-analysis of women who had IOL or PrlCS for “gestational-age window” in a twin pregnancy. The characteristics of these women were similar in terms of age, parity, gestational age at delivery, estimated fetal weight of each twin and antenatal steroid use. While the rate of the composite primary neonatal outcome was similar between the two sub-groups, adverse maternal outcome was found to be significantly lower with PrlCS in comparison with IOL (0% vs. 9.41%; *p* = 0.03; aOR 0.15; 95% CI 0–0.7). Again, this was mainly due to differences in the rates of hemorrhagic episodes (Table S1 & S2).

**Table 4:** Intention-to-treat analysis according to the original randomization groups for patient who required delivery when not in spontaneous labor

Outcome n (%)	Planned CS (*n* = 568)	Planned VD (*n* = 779)	aOR (95% CI)	*P* value
Composite primary outcome	22 (1.9%)	25 (1.6%)	0.82 (0.43–1.56)	0.55
Death	9 (0.8%)	12 (0.8%)	0.96 (0.35–2.64)	0.95
Serious neonatal morbidity^a^	13 (1.1%)	13 (0.8%)	0.72 (0.31–1.65)	0.45
Maternal death or serious maternal morbidity	54 (9.5%)	60 (7.7%)	0.79 (0.54–1.16)	0.24
Maternal death	1 (0.2%)	1 (0.1%)	0.72 (0.04–11.67)	0.82

*CS* Cesarean section, *VD* Vaginal delivery

Adjusted odds ratio (aOR) and their 95% confidence intervals (95%-CI) represent the result of a generalized estimating equation, accounting for maternal age, parity, previous CS, gestational age at delivery, presentation at delivery, antenatal corticosteroids use and for the correlation between infants from the same pregnancy

^a^Serious neonatal morbidity: birth trauma (long-bone fracture, other bone fracture, facial-nerve injury at 72 h of age or at discharge, intracerebral hemorrhage); Apgar score < 4 at 5 min; neurological (≥ 2 seizures before 72 h of age; coma; stupor or decreased response to pain); respiratory (assisted ventilation for ≥24 h by endotracheal tube, inserted before 72 h of age; bronchopulmonary dysplasia); neonatal sepsis before 72 h of age; necrotizing enterocolitis; grade III or IV intraventricular hemorrhage and cystic periventricular leukomalacia

## Discussion

The initial Twin Birth Study found no major differences in perinatal morbidity or mortality between planned vaginal delivery and planned cesarean section in women with twin gestations at 32^0/7^–38^6/7^ with the first twin in cephalic presentation [10]. A recent secondary analysis of the Twin Birth Study showed no change in neonatal or maternal outcomes between the study arms in women who presented with spontaneous labor [11]. In this secondary analysis we reviewed the outcomes of those women who did not have a spontaneous onset of labor, but had either IOL or PrlCS. While, IOL and PrlCS were found to have similar neonatal outcomes, PrlCS were associated with favorable maternal outcomes.

Analysis of this group of women who did not have a spontaneous onset of labor came with the difficulty of cross over between the original randomization groups. The high cross over rate from the original Twin Birth Study planned vaginal delivery arm to the PrlCS group may be partially explained by higher rate of non-cephalic presentation at delivery (either twin) found in the PrlCS group in compare to the IOL group. An increased risk for intra-partum CS following IOL in twins [8] is another possible factor affecting maternal decision to choose PrlCS over IOL. An earlier gestational age in delivery at the PrlCS group might represents higher risk fetuses with indications for delivery at an earlier stage of pregnancy. Hypothetically, higher risk fetuses are prone to increase rate of neonatal morbidity and mortality. Despite this, perinatal outcomes were comparable between the PrlCS and IOL groups, which may represent an overall more favorable result for PrlCS if the groups had been evenly matched in fetal risk level.

Our results correlate with the original Twin Birth Study regarding neonatal outcomes but differ in showing lower risk of adverse maternal outcomes in PrlCS, compared with IOL. The composite maternal outcome was higher in the IOL group mainly due to differences in hemorrhagic morbidity. This held true among the subset of women whose delivery was indicated due to “gestational-age window” in a twin pregnancy.

We are aware of the high rate of intra-partum CS in the IOL group, compared to what has been reported in singleton pregnancies following IOL [12, 13]. We postulate that a prior higher risk for CS in twins [14] in addition to late maternal age and increased rate of nulliparity (both having been associated with an intra-partum CS following IOL) can explain this finding [15, 16]. The increased maternal morbidity in the IOL group may be explained by the high rate of intra-partum CS in these women (41.3%).

There is minimal evidence regarding the relative safety of IOL versus CS of twins for women not in labor. Previous studies compared IOL in twins with IOL in singletons [7, 8], or compared IOL in twins with expectant management [17]. Our finding of fewer maternal adverse outcomes with CS is in contrast to studies performed by Drassinower et al. [18] and Ylilehto et al. [19]. Drassinower’s study of 1009 twin pregnancies found no significant difference in maternal hemorrhage or need for blood transfusion between cases of trial of labor or CS [18]. Similarly, Ylilehto et al., in a single center cohort study of 495 twin gestations, found fewer adverse maternal outcomes in the trial of labor group compared to elective CS group [19]. Of note, both studies included spontaneously laboring women and pre-labor women requiring IOL in the same group and had small study groups compared to the Twin Birth Study population. An argument can be made concerning safety of IOL in twins, and the various methods of IOL in twins. A secondary analysis by our group showed that there was no difference in maternal or fetal outcomes between methods of labor induction [20].

The strength of this secondary analysis lies in its size, multiple site recruitment and high rate of follow up on which to base results. It is a pioneer study to compare maternal outcomes in twin gestations that required delivery while not in labor. One might consider loss of intention to treat as a study weakness, as intention to treat analysis aims to give an unbiased estimate of treatment effect and preserves prognostic balance [21, 22]. Our results based on an intention-to-treat analysis found no differences in neonatal or maternal outcomes in the two planned mode of delivery groups. Nevertheless, due to the large cross over between the groups, intention-to-treat analysis in this case would unfairly include a large proportion of women, who in fact never attempted vaginal delivery, in the IOL group. The complication rates of their PrlCS could skew the data on both neonatal and maternal outcomes. As such, an analysis where attempted vaginal delivery (IOL) was compared to no attempt at vaginal delivery (PrlCS) gives results more akin to true life. This garners more representative results for clinical application and is beneficial when counseling patients with twins facing IOL. The main limitations of this study include the secondary analysis nature, not planned a priori, and therefore not powered to detect significant change between attempted modes of delivery in those who did not labor spontaneously. When performing a secondary analysis of a specific sub-population, using the same outcome, the practical meaning is that the results of this study are, by definition, underpowered. Lastly, neonatal complications found in this study may be impacted by confounding factors, such as gestational age at delivery or other obstetric complications, and not be exclusively related to mode of delivery.

## Conclusion

In twin pregnancies between 32^0/7^ and 38^6/7^ weeks of gestation who require delivery prior to onset of labor, our limited data suggests no fetal or neonatal benefit in either IOL or CS but possible maternal benefit with CS.

## Supplementary Information

**Additional file 1: Table S1.** Neonatal outcomes in women whose indication for delivery was “gestational-age window”.

**Additional file 2: Table S2.** Maternal outcomes in women whose indication for delivery was “gestational-age window”.

## Data Availability

The data that support the findings of this study are available from Twin Birth Study but restrictions apply to the availability of these data, which were used under license for the current study, and so are not publicly available. Data are however available from the authors upon reasonable request and with permission of Twin Birth Study.

## Abbreviations

CS: Cesarean section
IOL: Induction of labor
TBS: Twin Birth Study
VD: Vaginal delivery
PrlCS: Pre-labor cesarean section
OR: Odd ratios
CI: Confidence intervals
