# Supplementary material for: Cesarean delivery or induction of labor in pre-labor twin gestations: a secondary analysis of the twin birth study
Source: BMC Pregnancy Childbirth. 2020 Nov 17;20:702. doi: 10.1186/s12884-020-03369-x (PMC7672925; doi:10.1186/s12884-020-03369-x)
Supplement: Supplementary file 1 — Additional file 1: Table S1. Neonatal outcomes in women whose indication for delivery was “gestational-age window”. [file 12884_2020_3369_MOESM1_ESM.docx]

**Table S1.** Neonatal outcomes in women whose indication for delivery was “gestational-age window”.

| **Outcome n (%)** | **PrlCS (n==46)** | **IOL**  **(n=202)** | **aOR**  **(95% CI)** | **P Value** |
| --- | --- | --- | --- | --- |
| Composite primary outcome | 0 (0) | 9 (2.2) |  | 0.35 |
| Death | 0 (0) | 3 (0.8) |  | 0.99 |
| Serious neonatal morbidity | 0 (0) | 6 (1.5) |  | 0.36 |
| Birth trauma ^*^ | 0 (0) | 2 (0.5) |  | 0.99 |
| Apgar score <4 at 5 min | 0 (0) | 2 (0.5) |  | 0.99 |
| Abnormal level of consciousness † | 0 (0) | 0 (0.0) | N/E |  |
| ≥2 Seizures within 72 hr after birth | 0 (0) | 1 (0.3) |  | 0.99 |
| Assisted ventilation‡ | 0 (0) | 1 (0.3) |  | 0.99 |
| Neonatal sepsis within 72 hours after birth | 0 (0) | 0 (0) | N/E |  |
| Necrotizing enterocolitis | 0 (0) | 0 (0) | N/E |  |
| Cystic periventricular leukomalacia | 0 (0) | 0 (0) | N/E |  |

PrlCS- pre-labor cesarean section. IOL- induction of labor.

^¶^Serious neonatal morbidity: ^*^birth trauma (long-bone fracture, other bone fracture, facial-nerve injury at 72 hr of age or at discharge, intracerebral hemorrhage); Apgar score <4 at 5 minutes; neurological (≥ 2 seizures before 72 hours of age; coma; stupor or decreased response to pain); respiratory (assisted ventilation for ≥24 hours by endotracheal tube, inserted before 72 hours of age; bronchopulmonary dysplasia); neonatal sepsis before 72 hours of age; necrotizing enterocolitis; grade III or IV intraventricular hemorrhage and cystic periventricular leukomalacia.

^†^Abnormal level of consciousness: Coma, stupor or decreased response to pain, hyperalert, drowsy, or lethargic.

‡Assisted ventilation for ≥24 hr by means of endotracheal tube, inserted within 72 hr after birth.

Adjusted odds ratio (aOR) and their 95% confidence intervals (95%-CI) represent the result of a generalized estimating equation, accounting for maternal age, parity, previous CS, gestational age at delivery, presentation at delivery, antenatal corticosteroids use and for the correlation between infants from the same pregnancy.
