# Supplementary material for: Cesarean delivery or induction of labor in pre-labor twin gestations: a secondary analysis of the twin birth study
Source: BMC Pregnancy Childbirth. 2020 Nov 17;20:702. doi: 10.1186/s12884-020-03369-x (PMC7672925; doi:10.1186/s12884-020-03369-x)
Supplement: Supplementary file 2 — Additional file 2: Table S2. Maternal outcomes in women whose indication for delivery was “gestational-age window”. [file 12884_2020_3369_MOESM2_ESM.docx]

**Table S2.** Maternal outcomes in women whose indication for delivery was “gestational-age window”.

| **Outcome n (%)** | **PrlCS (n=46)** | **IOL**  **(n=202)** | **aOR**  **(95% CI)** | **P Value** |
| --- | --- | --- | --- | --- |
| Death or serious maternal morbidity  Death | 0 (0)  0 (0) | 19 (9.4)  1 (0.5) | 0.15 (0-0.7)  4.39 (0-83.4) | **0.03**  0.99 |
| Hemorrhage  Blood loss ≥1500 ml  Blood transfusion  D&C of uterus after delivery | 1 (2.2)  0 (0)  1 (2.2)  0 (0) | 17 (8.5)  6 (3.0)  13 (6.5)  2 (1.0) | 0.23 (0.0-1.8)  0.52 (0-2.8)  0.32 (0.0-2.5)  1.81 (0-15.3) | 0.17  0.58  0.28  0.99 |
| Laparotomy | 0 (0) | 0 (0) | - |  |
| Genital tract injury | 0 (0) | 0 (0) | - |  |
| Perineal third- or fourth-degree tear involving anal sphincter | 0 (0) | 1 (0.5) | 4.39 (0-83.4) | 0.99 |
| Thromboembolism requiring anticoagulant therapy | 0 (0) | 0 (0) | - |  |
| Infection, excluding wound infection | 0 (0) | 2 (1.0) | 1.81 (0-15.3) | 0.99 |
| Wound infection^*^ | 0 (0) | 3 (1.5) | 1.13 (0-7.6) | 0.99 |
| Wound dehiscence or breakdown | 0 (0) | 1 (0.5) | 4.39 (0-83.4 | 0.99 |

PrlCS- pre-labor cesarean section**.** IOL- induction of labor.

^*^D&C- Dilation and curettage.

× Genital Tract Injury: Need for hysterectomy; vulvar or perineal hematoma requiring evacuation; broad-ligament hematoma confirmed by means of ultrasonography, CT, or MRI; intraoperative damage to the bladder, ureter, or bowel requiring repair; fistula involving the genital tract.

^†^Wound infection: Infection requiring prolongation of hospital stay, infection requiring readmission to hospital, infection requiring repeated treatment as an outpatient.

Adjusted odds ratio (aOR) and their 95% confidence intervals (95%-CI) represent the result of a generalized estimating equation, accounting for maternal age, parity, previous CS, gestational age at delivery, presentation at delivery, antenatal corticosteroids use and for the correlation between infants from the same pregnancy.
